# Novel connections and physical implications of thermal metamaterials with imperfect interfaces

**DOI:** 10.1038/s41598-022-06719-1

**Published:** 2022-02-17

**Authors:** Tungyang Chen, Jun-Hong Lin

**Affiliations:** Department of Civil Engineering, National Cheng Kung University, Tainan, 70101 Taiwan, ROC

**Keywords:** Applied physics, Engineering, Mathematics and computing

## Abstract

Thermal metamaterials are of great importance in advanced energy control and management. Previous studies mainly focused on interfaces with perfect bonding conditions. In principle, imperfectness always exists across interface and the effect is intriguingly important with small-length scales. This work reports the imperfect interface effect in thermal metamaterials thoroughly. Low conductivity- and high conductivity-type interfaces are considered. We show that an object can always be made thermally invisible, with the effect of imperfect interface, as that of a homogeneous background material. This unprecedented condition is derived in an exact and analytic form, systematically structured, with much versatile and physical implications. Conditions for thermal shielding and enhancements are analytically found and numerically exemplified, highlighting the specific role of material and geometric parameters. We find that both types of interfaces are complementing with each other which, all together, will constitute a full spectrum to achieve the thermal invisibility. The analytic finding offers a general perception that adds to the understanding of heat transport mechanism across interfaces in thermal metamaterials, in ways that drastically distinct from that of ideal interfaces. This finding opens up new possibilities for the control and management of thermal metamaterials with imperfect bonding interfaces.

## Introduction

Management and control of heat is familiar to all of us and is ubiquitous in our daily life. The phenomena of heat transfer across solid interfaces between different materials are of central importance in modern technologies and applications, particularly with scaling down of the devices^1^. On the other hand, thermal devices that are capable of confine energy, preferably with an enhanced intensity, are becoming imperative especially in the field of energy harvesting. To realize these functionalities in various applications, it would require an in-depth understanding on the transfer mechanism across interfaces^2^, particularly counting into the effect of bonding imperfectness.

In the last decade, significant progress was made in the compositions of materials, referred to as thermal metamaterials, to control the flow of heat along a certain path. Among them, by surrounding an artificially designed material, it is possible to make an object undetectable from thermal measurements^3,4^. The coordinate transformation method^5^, originally proposed in optics for invisibility cloaks, offers a systematic approach capable of precisely manipulating wave-based as well as diffusion-based phenomena^6^ in a desired manner. Han et al.^7^ showed that a circular anisotropic layer can serve as a tunable device to control thermal energy within a certain area. Other relevant works on thermal devices have been studied theoretically and numerically^8–12^. Substantial progress has also been made in experimental verifications of thermal devices^13–23^. For a general exposition of the subject, one can refer to the review articles^24–28^ and the monograph^29^. All these aforementioned studies mainly consider ideal interfaces. In principle, imperfectness of bonding always exists across the interface between two dissimilar solids in contact. Particularly, in small-length scales, an ideal interface framework could not be adequate and that interfacial thermal contact will become non-negligible^2,30^. For a given volume fraction, the smaller the partcle size, the larger will be the total contact surface area between different materials. Yet very little work focused on the effects of imperfect interface effect, nor on the general transport mechanisms of thermal energy across interfaces of thermal metamaterials. Zheng and Li^31^ recently demonstrated that, with the interfacial thermal resistance, the temperature field outside the cloak could be greatly distorted, in contrast to that of ideal interfaces.

In this study, we consider steady-state heat conduction, under a macroscopic scale, governed by Fourier’s law. We examine the effect of imperfect interface in the design of a homogeneous thermal metamaterial, mostly in analytic forms, highlighting the specific role of material and geometric parameters. We show that, with the imperfect bonding across interfaces, under a certain universal connection, an object can still be made thermally invisible as that of a homogeneous material. This connection, referred to as thermal invisibility condition, can be exactly constructed. The underlying theory is to utilize the neutral inclusion concept in mechanics of composites^32^, which allows us to build the invisibility condition through a series of homogenization process. Two different types of imperfect interfaces, low conductivity—(LC) and high conductivity—(HC) types, are considered. The LC-type interface exhibits an interfacial thermal resistance^30^, where when a heat flux passes through an interface, a proportional temperature jump will occur. On the other hand, a HC-type interface^33^ is physically opposite to that of LC-type interface. In this kind of interface the temperature field is continuous across the interface, while the jump of the normal component of heat flux is proportional to the surface Laplacian of the temperature field at the contact boundary. Unlike the simple condition for perfect bonding situations, the thermal invisibility conditions now exhibit size effects, but have well-structured forms, fully elucidatory from the viewpoints of effective conductivity of composite cylinders assemblages^32^. Quite a few previously known results can be deduced as simplified solutions. It appears that the field responses associated with LC- and HC-type interfaces are exactly linked with each other, which implies that, irrespective of the material and geometric parameters, the LC- and HC-type interfaces, all together, will constitute a full spectrum to achieve the thermal invisibility condition. Conditions of thermal shielding and concentrating are analytically found. In general, the imperfect interfaces will broadly affect the energy flow across the interface, and that significant factors dominating the concentrating or shielding behavior are identified from the analytic result.

It is mentioned that thermal contact resistance could be due to poor mechanical contact and roughness at their common boundaries, and also due to the different physical properties in different materials^31^. As demonstrated by Sanchez-Palencia^34^, the LC- and HC-type interface can be derived from the configuration of a thin interphase layer of relatively low or high conductivity. A mathematical derivation of the interface conditions can be found in Benveniste and Miloh^35^. Apart from the LC- and HC-type interfaces, we mention that there is a third model, referred to as the general imperfect interface model^36,37^, in which both the temperature and the normal heat flux could have possible jumps across the interface. This model is to simulate, in an asymptotic manner, the three-phase medium as a two-phase medium but with a general imperfect interface condition. The original exposition of this general interface model was first proposed by Bovik^38^ in the context of dynamic elasticity and acoustics.

We start from a summary of the main results, followed by a detailed exposition of the analytic thermal invisibility condition. Numerical illustrations as well as finite-element simulations will be presented to highlight the interplay for the balance among the material and geometric parameters. At present, studies on the interface bonding effects of thermal metamaterials are rather scarce and the current understanding on the effect of imperfect bonding in thermal metamaterials remains elusive, partly due to the complexity of the interface condition that obstructs a general image. The present analytic studies in this way shed light on this. Since the interface in general may not be perfect, the finding could provide a general guideline that facilitates the design and experiment of homogeneous thermal metamaterials.

## Results

At a macroscale, heat conduction can be viewed as a diffusion process in which thermal energy flows from hot region to cold region. The heat equation, described by Fourier’s law and the conservation of energy, characterizes the temperature field over space and time. Under a steady state condition, in the absence of heat source, the temperature field in a medium is governed by the Fourier law $\nabla \cdot \left( {\mathbf{k} \nabla T} \right) = 0$, where *T* is the temperature field and **k** is the thermal conductivity tensor. For natural materials the values of thermal conductivity vary a great deal, ranging from poorly conducting materials with *k* ≈ 0.02 W m^−1^ K^−1^ to good conductors with *k* ≈ 400 W m^−1^ K^−1^.

**Figure 1:**
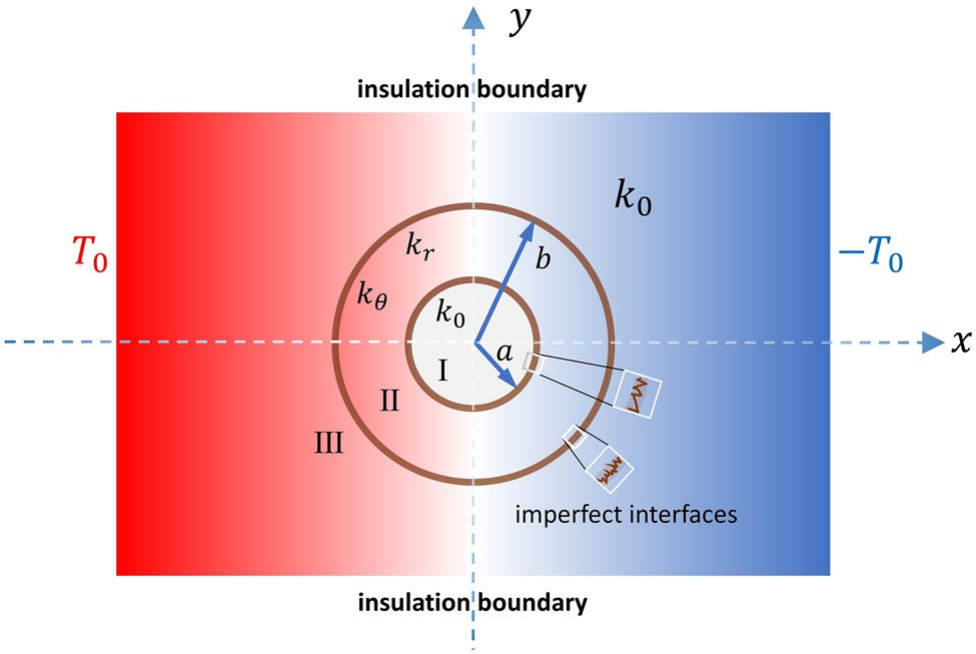
A schematic illustration of a circular thermal device with imperfect interfaces. Region I has an isotropic conductivity *k* = *k*_0_ and is the targeted region for heat flux control. Region II is the designed region with constant radial and circumferential conductivities. Region III is the surrounding background material with the same isotropic conductivity as in Region I. The interfaces at *r* = *a* and *r* = *b* are imperfectly bonded with different interface parameters.

We consider a two-dimensional model depicted as in Fig. 1. Region I is isotropic with conductivity *k* = *k*_0_, which is the region for heat flux control. Region II is the thermal metamaterial made of an anisotropic layer with constant radial and circumferential conductivities given as

1 $${\mathbf{k}} = \left( {\begin{array}{*{20}l} {k_{rr} } & 0 \\ 0 & {k_{\theta \theta } } \\ \end{array} } \right).$$

Region III is the surrounding background material with the same isotropic conductivity *k* = *k*_0_ as in Region I. Along the *x*-direction, a uniform thermal intensity *E* is prescribed. The interfaces between Region I and Region II, and between Region II and Region III, could be imperfectly bonded, with either LC- or HC-type. We will show that a tailored set of constant materials and geometric parameters in Region II can act as a functional thermal metamaterial in such a way that the outer field in Region III will remain to have a constant temperature intensity *E*, as if the whole medium is spatially homogeneous. In addition, the thermal intensity in Region I can be tuned to be greatly enhanced or shielded.

### Low conductivity-type interface

The LC-type interface is that the interface exhibits thermal contact resistance, or Kapitza resistance^30^, between the two surfaces in contact. In this case, the temperature will have a drop across the interface, while the normal component of heat flux ${\mathbf{q}} \cdot {\mathbf{n}} = - \left( {{\mathbf{k}}\nabla T} \right) \cdot {\mathbf{n}}$ will be continuous. For theoretical and numerical studies in the context of composites, we mention the works of Refs.^39–43^. Specifically, the interface conditions for the LC type of interface at *r* = *a* are

2 $$k_{r} \frac{{\partial T_{2} }}{\partial n} = k_{0} \frac{{\partial T_{1} }}{\partial n}\left. { = \beta_{a} \left( {T_{2} - T_{1} } \right)} \right|_{r = a} .$$

The interface condition at $r = b$ will be given in Supplementary Material S3 for brevity. Here $\partial /\partial n$ is the normal derivative at the interfaces and *T*_1_, *T*_2_ and *T*_3_ represent the temperature fields in Regions I, II and III, respectively. The interface parameter *β* is defined as the ratio between the temperature drop and the heat flow across the interface, with a dimension of W m^−2^ K^−1^. When $\beta \to \infty$, the LC-type interface will reduce to that of the perfect bonding interface. We shall use *β*_*a*_ to denote the LC-type interface parameter at interface *r* = *a*, and similarly *β*_*b*_ for *r* = *b*. It should be emphasized that the mathematical framework of LC-type interface can be formulated by considering a thin interphase layer, of a constant thickness *t* with relatively low conductivity *k*_*c*_, compared to those of the two neighboring regions^39^. By taking an asymptotic analysis with equilibrium considerations, one can show that the effect of the interphase is equivalent to an interface between the two phases with a LC-type of interface condition, where $\beta = \mathop {\lim }\limits_{{t \to 0,k_{c} \to 0}} k_{c} /t$. We will show that, in the presence of thermal contact resistances, it is possible to achieve the thermal invisibility in the outer Region III. The exact connection for the LC-type can be exactly determined as

3 $$g\frac{{\left[ {g + \left( {1 + \tilde{\beta }_{a} } \right)} \right] - c^{\lambda } \left[ {g - \left( {1 + \tilde{\beta }_{a} } \right)} \right]}}{{\left[ {g + \left( {1 + \tilde{\beta }_{a} } \right)} \right] + c^{\lambda } \left[ {g - \left( {1 + \tilde{\beta }_{a} } \right)} \right]}} + \tilde{\beta }_{b} = 1,{\text{ for }}g < 1,$$

where *c* = (*a/b*)^2^ is the area fraction of Region I, *g* = *k*_0_/*k*_*G*_, $\tilde{\beta }_{a} = {{k_{0} } \mathord{\left/ {\vphantom {{k_{0} } {\left( {a\beta_{a} } \right)}}} \right. \kern-\nulldelimiterspace} {\left( {a\beta_{a} } \right)}}$ and $\tilde{\beta }_{b} = {{k_{0} } \mathord{\left/ {\vphantom {{k_{0} } {\left( {b\beta_{b} } \right)}}} \right. \kern-\nulldelimiterspace} {\left( {b\beta_{b} } \right)}}.$ The quantity $k_{G} = \sqrt {k_{r} k_{\theta } }$ is the geometric average of *k*_*r*_ and *k*_*θ*_, and $\lambda = \sqrt {k_{\theta } /k_{r} }$ is a parameter that signifies the anisotropy of the conductivity tensor in Region II. Apparently, *λ* = 1 stands for an isotropic material in Region II. We note that all the parameters in (3) are non-dimensional. Here the top tilde of $\tilde{\beta }$ is used for the non-dimensional interface parameter with LC-type interfaces, while a top hat will be used for those of HC-type interfaces to be described later. The constant *g* is a parameter indicating the relative magnitude of *k*_*G*_ and *k*_*0*_. For example, *g* < 1 means *k*_0_ < *k*_*G*_, whereas *g* > 1 indicates *k*_*0*_ > *k*_*G*_. Note that *g* = 1 is exactly the thermal neutrality condition for the ideal interfaces. In our formulation, we consider that both the interfaces at *r* = *a* and *r* = *b* are imperfectly bonded with different interface parameters. When only one interface is imperfect, we can simply let $\tilde{\beta }_{a} = 0$ or $\tilde{\beta }_{b} = 0$ in (3) and directly deduce a simplified version of (3). Particularly, when the thermal invisibility condition (3) is fulfilled, the temperature field in the Region III will remain as $T_{3} = - Er\cos \theta .$ This suggests that Regions I and II, togther with the effect of the imperfect interfaces, are effectively equivalent to that of a homogenous circular cylinder with conductivity *k*_0_. We note that, in addition to the interface parameters $\tilde{\beta }_{a}$ and $\tilde{\beta }_{b}$, the thermal invisibility condition (3) also depend on three parameters, *g* = *k*_0_/*k*_*G*_, *λ* and *c* = (*a/b*)^2^. Note that in the case of perfect bonding interfaces, we have $\beta_{a} \to \infty$,$\beta_{b} \to \infty$, that is $\tilde{\beta }_{a} \to 0$, $\tilde{\beta }_{b} \to 0$. Equation (3) will exactly reduce to the effective conductivity of composte cylinder assemblage^44^, an isotropic inclusion of conductivity *k*_0_ coated with a cylindrically orthotropic layer with conductivity tensor (1). It can be verified analytically, by direct expansion, that the thermal invisibility condition (3) will recover exactly to the known neutrality condition for the perfect bonding situation^7^, $k_{rr} k_{\theta \theta } = k_{0}^{2}$. Note that this latter connection under the perfect bonding condition is independent of the absolute sizes of the regions I and II, nor of the area fraction. Physically, the LC-type interface, compared to that of perfect interface, exhibits a thermal contact resistance at interface. Thereby, in the presence of a positive interface parameter *β*, to fulfill the thermal invisibility condition, one would necessarily have *k*_*G*_ > *k*_0_, i.e., g < 1.

### High conductivity-type interface

**Figure 2:**
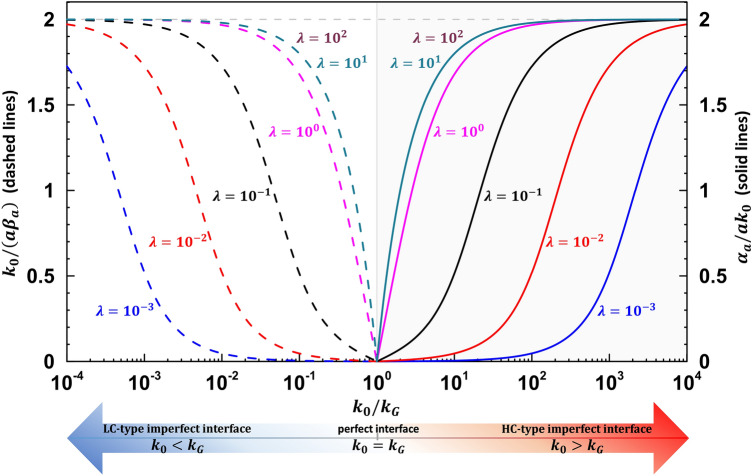
Numerical illustrations of the geometric and material parameters that fulfill the thermal invisibility conditions (3) and (5) for *c* = 1/4. The horizontal axis is *k*_0_/*k*_*G*_, expressed in logarithmic scale. The left panel, corresponding to the range of *k*_0_/*k*_*G*_ < 10^0^, will invoke a LC-type interface parameter *k*_0_*/aβ*_*a*_. Different curves (dashed lines) correspond to different values of *λ*. The right panel, corresponding to the range of *k*_0_*/k*_*G*_ > 10^0^, will involve a HC-type interface parameter $\alpha_{a} /ak_{0} .$ Different curves (solid lines) corresponds to different values of *λ* and the vertical axis is marked on the right-side. Note that the curve *λ* = 10^0^ corresponds to an isotropic material in Region II in which $k_{r} = k_{\theta } .$

The behavior of HC-type interface is physically opposite to that of LC-type interface. The mathematical framework of HC-type interface can be derived by considering a thin interphase layer of a constant thickness *t* with relatively high conductivity *k*_*c*_. Related studies on the effect of HC-type interfaces in composite systems can be found, for example, in the papers^33,45–49^. In the context of elasticity, HC-type interface is relevant to the interface conditions with surface stress effects^50^. Specifically, the HC-type interface conditiond at *r* = *a* are

4 $$\left. {T_{1} = T_{2} } \right|_{r = a} ,\;\left. {\left( {k_{0} \frac{{\partial T_{1} }}{\partial r} - k_{r} \frac{{\partial T_{2} }}{\partial r}} \right)} \right|_{r = a} = \left. {\left( {\alpha_{a} \Delta_{s} T} \right)} \right|_{r = a} ,$$

where $\Delta_{s}$ is the surface Laplacian operator. The interface condition at *r* = *b* is omitted here and is expressed in (S4) for conciseness. The parameter of the HC-type interface can be derived as $\alpha = \mathop {\lim }\limits_{{t \to 0,k_{c} \to \infty }} k_{c} t$, where *α* has a dimension of W K^−1^. We note that $\alpha_{a} = \alpha_{b} = 0$ will correspond to ideal interface conditions. For the configuration of Fig. 1, we find that, in the presence of HC-type interfaces, when the material and geometric parameters fulfill the condition

5 $$g^{ - 1} \frac{{\left[ {g\left( {1 + \hat{\alpha }_{a} } \right) + 1} \right] + c^{\lambda } \left[ {g\left( {1 + \hat{\alpha }_{a} } \right) - 1} \right]}}{{\left[ {g\left( {1 + \hat{\alpha }_{a} } \right) + 1} \right] - c^{\lambda } \left[ {g\left( {1 + \hat{\alpha }_{a} } \right) - 1} \right]}} + \hat{\alpha }_{b} = 1,{\text{ for }}g > 1,$$

the temperature field in Region III will remain to have $T_{3} = - Er\cos \theta$, as if the whole medium is homogeneous. In (5), we have defined the dimensionless parameters $\hat{\alpha }_{a} = {{\alpha_{a} } \mathord{\left/ {\vphantom {{\alpha_{a} } {\left( {ak_{0} } \right)}}} \right. \kern-\nulldelimiterspace} {\left( {ak_{0} } \right)}}$, $\hat{\alpha }_{b} = {{\alpha_{b} } \mathord{\left/ {\vphantom {{\alpha_{b} } {\left( {bk_{0} } \right)}}} \right. \kern-\nulldelimiterspace} {\left( {bk_{0} } \right)}}$. Note again, as in (3), in addition to the interface parameters $\hat{\alpha }_{a}$ and $\hat{\alpha }_{b}$, the invisibility condition (5) also depend on the three parameters, *g*, *λ* and the area fraction *c*. Note also that for perfect bonding interfaces we have $\alpha_{a} \to 0$, $\alpha_{b} \to 0$, namely $\hat{\alpha }_{a} \to 0$ and $\hat{\alpha }_{b} \to 0.$ It can be shown that Eq. (5) will recover the known condition under the perfect bonding conditions^7^ $k_{rr} k_{\theta \theta } = k_{0}^{2}$. Physically, the HC-type interface represents a more conductive behavior than that of the perfect bonding interface. As such, when we have *k*_0_ > *k*_*G*_, to fulfill the invisibility condition it is necessary to have HC-type interfaces to balance the mismatch between *k*_0_ and *k*_*G*_. To address the point, in Fig. 2 we plot the relation between *k*_0_/*k*_*G*_ and the dimensionless interface parameters, $\tilde{\beta }_{a}$ or $\hat{\alpha }_{a}$. In the numerical illustrations, we have assumed *β*_*a*_ = *β*_*b*_ = *β* and *α*_*a*_ = *α*_*b*_ = *α*, as both interfaces separate Region II and a region with isotropic conductivity *k*_*0*_. For numerical illustrations, we specify *c* = (*a/b*)^2^ = 1/4. Note that the horizontal axis is expressed in logarithmic scale. The left panel of Fig. 2 corresponds to the range of *k*_0_/*k*_*G*_ < 10^0^, where a LC-type interface is needed to compensate the conductivity of Region II so that the outer field in Region III will remain unaffected.

**Figure 3:**
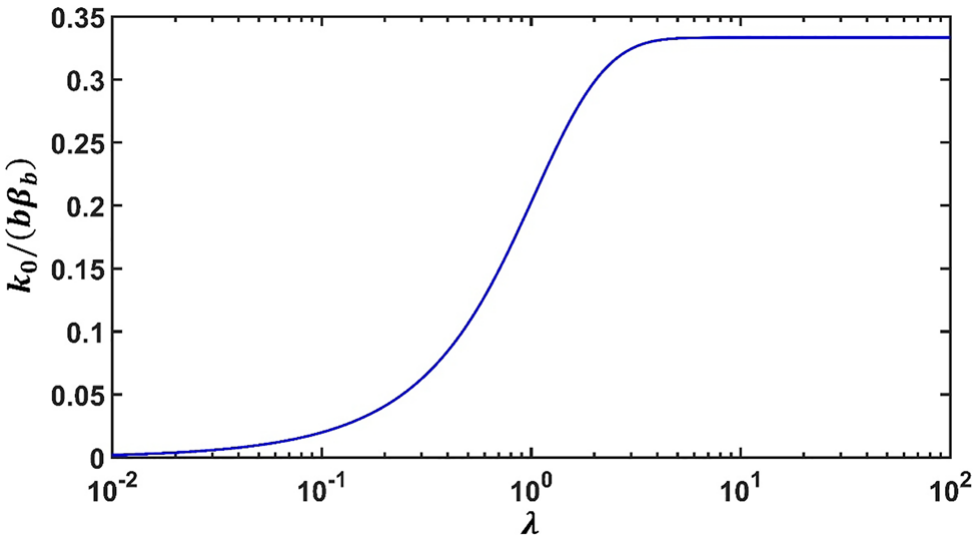
The value of $\tilde{\beta }_{b} = {{k_{0} } \mathord{\left/ {\vphantom {{k_{0} } {b\beta_{b} }}} \right. \kern-\nulldelimiterspace} {b\beta_{b} }}$ versus the anisotropy parameter *λ*. In this illustration, we specify *g* = 2/3, *c* = 1/4 and *b* = 1 μm. Note the curve also applies to the case of *g* = 3/2, but the vertical axis is now changed to the scale of $\hat{\alpha }_{a}$.

The associated dimensionless interface parameter *k*_0_/*aβ* is indicated on the vertical axis on the left panel. On the other hand, when *k*_0_/*k*_*G*_ > 10^0^, a HC-type interface will be invoked to render the outer field invisible, as that of a homogeneous medium. The corresponding non-dimensional interface parameter *α/ak*_0_ is labelled on the vertical axis on the right side. Different curves for various values of *λ* are plotted. The curves corresponding to *λ* = 10 and *λ* = 10^2^ are nearly overlapping, and thus for *λ* > 10^2^ the corresponding curve can be viewed as an asymptotic limit. We note that all curves are intersecting at a single point *k*_*G*_*/k*_0_ = 10^0^, which is the situation for perfect bonding interfaces corresponding to $\alpha \to 0$ or $\beta \to \infty .$ Interestingly, the curves between the two sides along the line *k*_0_/*k*_*G*_ = 10^0^ appear to possess a reflection symmetry. We will confirm this later in “Methods” section. In Fig. 3, we plot the value of $\tilde{\beta }_{b} = {{k_{0} } \mathord{\left/ {\vphantom {{k_{0} } {b\beta_{b} }}} \right. \kern-\nulldelimiterspace} {b\beta_{b} }}$ versus the anisotropy parameter *λ* for the case of *g* = 2/3, *c* = 1/4 and *b* = 1 μm. It is seen that when *λ * → 0, we have $\tilde{\beta }_{b} \to 0$. On the other hand, when *λ * → ∞, we have $\tilde{\beta }_{b} \to {1 \mathord{\left/ {\vphantom {1 3}} \right. \kern-\nulldelimiterspace} 3}$. Also we observe when *λ* < 10^−2^, or *λ* > 10^−2^, the curves are virtually unchanged. Note Fig. 3 also applies to the case of *g* = 3/2, but now the vertical axis needs to be replaced by the scale of $\hat{\alpha }_{b}$.

In short, the exact thermal invisibility conditions are expressed in analytical forms in Eq. (3) for the LC-type and in Eq. (5) for the HC-type interfaces. The explicit expressions depend on the material properties as well as the geometric parameters. Note that all these parameters are non-dimensional, including the interface parameters $\tilde{\beta }$ and $\hat{\alpha }$, the anisotropic ratio $\lambda = \sqrt {{{k_{\theta } } \mathord{\left/ {\vphantom {{k_{\theta } } {k_{r} }}} \right. \kern-\nulldelimiterspace} {k_{r} }}}$, the ratio *g* = *k*_0_/*k*_*G*_ and the area fraction *c* = (*a/b*)^2^. A detailed study on the effects of extreme values of $\lambda$, *g* = *k*_0_/*k*_*G*_ and *c* = (*a/b*)^2^ is given in Supplementary Materials. This will provide theoretical examinations, in complement with the numerical illustrations given in Figs. 2 and 3, on the trend under the extreme values of *g* = *k*_0_/*k*_*G*_, $\lambda$ and *c* to achieve the thermal neutrality conditions (3) or (5). Both theoretical and numerical results agree perfectly under extreme values of *g* for Fig. 2 and $\lambda$ for Fig. 3.

## Theoretical analysis

**Figure 4:**
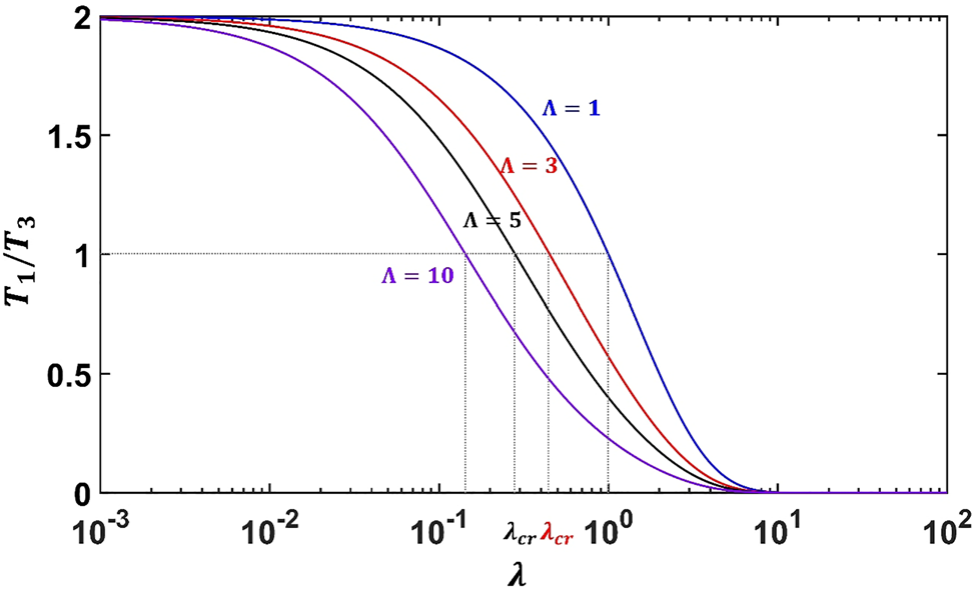
The ratio of *T*_1_*/T*_3_ versus *λ* for different values of Λ. The area fraction *c* for the numerical illustration is specified as *c* = 1/4. Note that the figure will be identical to that of the HC-type interface if Λ is replaced by Γ.

We now present the theoretical analysis of the conduction of the geometric model shown in Fig. 1. The governing temperature fields for Regions I and III are simply the Laplace equation, while the field equation for Region II can be written as $\frac{{\partial^{2} T}}{{\partial r^{2} }} + \frac{1}{r}\frac{\partial T}{{\partial r}} + \frac{{\lambda^{2} }}{{r^{2} }}\frac{{\partial^{2} T}}{{\partial \theta^{2} }} = 0.$ Boundary condition $T\left( {x = r\cos \theta = \pm x_{0} } \right) = \mp T_{0}$ are prescribed on the left and right surfaces of the configuration (Fig. 1), with $T_{0} = - Ex_{0}$ and *E* is the prescribed constant intensity. Taking into account the boundary condition and the geometric symmetry, the temperature potentials in the three regions are given in (S2). If the medium is homogeneous throughout, the temperature gradient will be spatially uniform. But in the presence of the Region II together with the imperfect interfaces along *r* = *a* and *r* = *b*, heat flows will be generally distorted. We find that under the condition of (3) or (5), the outer field in Region III will remain to possess a uniform intensity *E*, as if the Region II and the imperfect interfaces are entirely invisible. A direct outline of the solution procedure will be given in Supplementary Material. In “Methods” section, instead, a simple exposition of the thermal invisibility condition based on the neutral inclusion concept is presented through a series of homogenization process (see Fig. 6). Now under the invisibility condition (3), we find that the temperature field in Regions I and II can be exactly expressed as

6 $$\left. {\frac{{T_{1} }}{{T_{3} }}} \right|_{\beta } = \frac{{c^{{ - {1 \mathord{\left/ {\vphantom {1 2}} \right. \kern-\nulldelimiterspace} 2}}} }}{{\cosh \left( {\frac{\lambda }{2}\ln c} \right)\left( {1 - \frac{{\left( {1 + \tilde{\beta }_{a} } \right)}}{g}\tanh \left( {\frac{\lambda }{2}\ln c} \right)} \right)}},$$

7 $$\left. {\frac{{T_{2} }}{{T_{3} }}} \right|_{\beta } = g\frac{b}{r}\frac{{\cosh \left( {\lambda \ln \frac{r}{a}} \right)\left( {\tanh \left( {\lambda \ln \frac{r}{a}} \right) + \frac{{\left( {1 + \hat{\beta }_{a} } \right)}}{g}} \right)}}{{\cosh \left( {\frac{\lambda }{2}\ln c} \right)\left( {1 - \frac{{\left( {1 + \hat{\beta }_{a} } \right)}}{g}\tanh \left( {\frac{\lambda }{2}\ln c} \right)} \right)}},$$

where $T_{3} = - Er\cos \theta$. Here the subscript $\beta$ on the left-hand side is used to signify the fields for LC-type interfaces, in distinction with that of HC-type interfaces to be presented later. We are particularly interested with the ratio of *T*_1_*/T*_3_, in which a value of *T*_1_*/T*_3_ > 1 will represent a concentrating effect, while a value of *T*_1_*/T*_3_ < 1 will indicate a cloaking or shielding effect. We first note that when *c* → 0, that is $a \ll b$, we have *T*_1_*/T*_3_ → 0. On the other hand, when *c * →  1, that is *a* ≈ *b*, we have *T*_1_*/T*_3_ → 1. Since 0 < *c* ≤ 1, it is evident that $- \infty < \ln c \le 0$ and $1 \le c^{{ - {1 \mathord{\left/ {\vphantom {1 2}} \right. \kern-\nulldelimiterspace} 2}}} = {b \mathord{\left/ {\vphantom {b a}} \right. \kern-\nulldelimiterspace} a} < \infty$. Also, from elementary algebra, we know that $\cosh \left( {\frac{\lambda }{2}\ln c} \right) \ge 1$ and $- 1 < \tanh \left( {\frac{\lambda }{2}\ln c} \right) < 0$. Thus, in view of (6), we conclude that the constant intensity in Region I is always bounded by the ratio *b/a*,

8 $$\left. {\frac{{T_{1} }}{{T_{3} }}} \right|_{\beta } < \frac{{{b \mathord{\left/ {\vphantom {b a}} \right. \kern-\nulldelimiterspace} a}}}{{\underbrace {{\cosh \left( {\lambda \ln \frac{a}{b}} \right)}}_{ \ge \, 1}\underbrace {{\left( {1 + \frac{{\left( {1 + \tilde{\beta }_{a} } \right)}}{g}} \right)}}_{ \ge \, 1}}} \le \frac{b}{a},$$

where the upper limit is attained by the perfect bonding conditions, that is *g* = 1,$\tilde{\beta }_{a} = \tilde{\beta }_{b} = 0$, and *λ*  → 0. Note that for perfect bonding interfaces, the internal fields will be exactly reduced to the known solutions ${{T_{1} } \mathord{\left/ {\vphantom {{T_{1} } {T_{0} = \left( {{a \mathord{\left/ {\vphantom {a b}} \right. \kern-\nulldelimiterspace} b}} \right)}}} \right. \kern-\nulldelimiterspace} {T_{0} = \left( {{a \mathord{\left/ {\vphantom {a b}} \right. \kern-\nulldelimiterspace} b}} \right)}}^{\lambda - 1}$ and ${{T_{2} } \mathord{\left/ {\vphantom {{T_{2} } {T_{0} = \left( {{r \mathord{\left/ {\vphantom {r b}} \right. \kern-\nulldelimiterspace} b}} \right)}}} \right. \kern-\nulldelimiterspace} {T_{0} = \left( {{r \mathord{\left/ {\vphantom {r b}} \right. \kern-\nulldelimiterspace} b}} \right)}}^{\lambda - 1}$^7^. To illustrate the variation of intensity in Region I, we see that the constant intensity in (6) will depend on *c*, *λ*, and ${{\left( {1 + \tilde{\beta }_{a} } \right)} \mathord{\left/ {\vphantom {{\left( {1 + \tilde{\beta }_{a} } \right)} g}} \right. \kern-\nulldelimiterspace} g}$. Note these three parameters need to follow the thermal invisibility condition (3). For convenience, let us define $\Lambda = {{\left( {1 + \tilde{\beta }_{a} } \right)} \mathord{\left/ {\vphantom {{\left( {1 + \tilde{\beta }_{a} } \right)} g}} \right. \kern-\nulldelimiterspace} g}$ and rewrite (3) as in Eq. (S5). In Fig. 4, we plot the value of *T*_1_*/T*_3_ versus *λ* for different values of Λ, where the area fraction *c* is specified as $c = 1/4$. Since $\tilde{\beta }_{a} \ge 0$ and 0 < *g* < 1, the value of Λ is always greater than 1 and that Λ = 1 corresponds to the perfect bonding condition. If one defines $\lambda_{cr}$ as the value of *λ* such that *T*_1_*/T*_3_ = 1, it can be seen from Fig. 4 that, for a fixed value of *c*, when the value of Λ is increased, a smaller value of *λ* is needed to attain the value *T*_1_*/T*_3_ = 1.

Next, we consider the HC-type interfaces. Now under the condition (5), we find that the temperature field in the Regions I and II can be exactly expressed as

9 $$\left. {\frac{{T_{1} }}{{T_{3} }}} \right|_{\alpha } = \frac{{{b \mathord{\left/ {\vphantom {b a}} \right. \kern-\nulldelimiterspace} a}}}{{\cosh \left( {\frac{\lambda }{2}\ln c} \right)\left( {1 - g\left( {1 + \hat{\alpha }_{a} } \right)\tanh \left( {\frac{\lambda }{2}\ln c} \right)} \right)}},$$

10 $$\left. {\frac{{T_{2} }}{{T_{3} }}} \right|_{\alpha } = \frac{b}{r}\frac{{\cosh \left( {\lambda \ln \frac{r}{a}} \right)\left( {1 + g\left( {1 + \hat{\alpha }_{a} } \right)\tanh \left( {\lambda \ln \frac{r}{a}} \right)} \right)}}{{\cosh \left( {\frac{\lambda }{2}\ln c} \right)\left( {1 - g\left( {1 + \hat{\alpha }_{a} } \right)\tanh \left( {\frac{\lambda }{2}\ln c} \right)} \right)}}.$$

We note that the two terms in the denominator, as in (6) and (7), are always greater than unity, thus it is seen that *T*_1_*/T*_3_ ≤ *b/a*. The upper limit of is achieved for *λ*  → 0 under the perfect bonding condition, that is *g* = 1 and $\hat{\alpha }_{a} = \hat{\alpha }_{b} = 0$. Again, the expressions (9) and (10) can be shown to reduce to the known solution for perfect bonding interfaces. For simplicity, we can define $\Gamma = g\left( {1 + \hat{\alpha }_{a} } \right)$ and rewrite the thermal invisibility condition (5) as in (S6). The value of $\Gamma$ is always greater than 1, since $\hat{\alpha }_{a} \ge 0$ and *g* > 1, and that Γ = 1 corresponds to the perfect bonding situation. For a fixed value of *c*, one can draw diagrams for the value of $\left. {{{T_{1} } \mathord{\left/ {\vphantom {{T_{1} } {T_{3} }}} \right. \kern-\nulldelimiterspace} {T_{3} }}} \right|_{\alpha }$ versus *λ* for different values of $\Gamma .$ Note that the illustration for HC-type interfaces will be exactly the same with that of the LC-type interface simply by replacing Λ by Γ, which will be explained later in “Methods” section.

**Figure 5:**
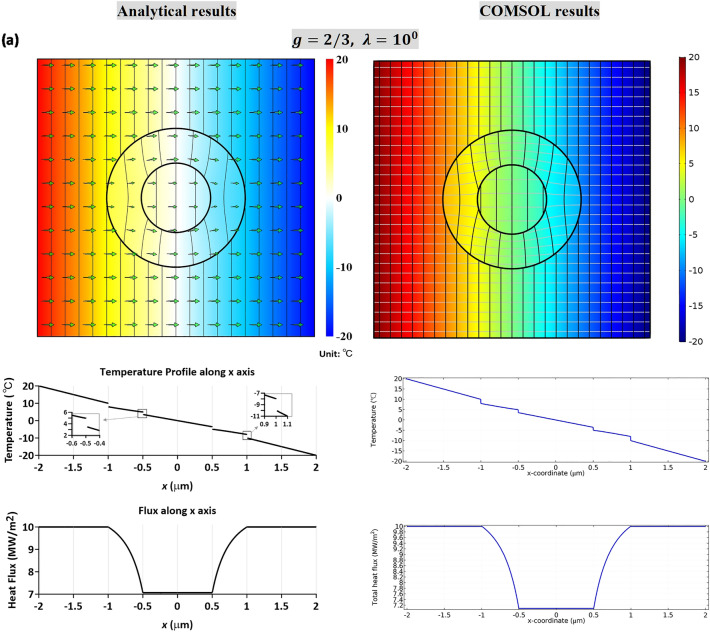

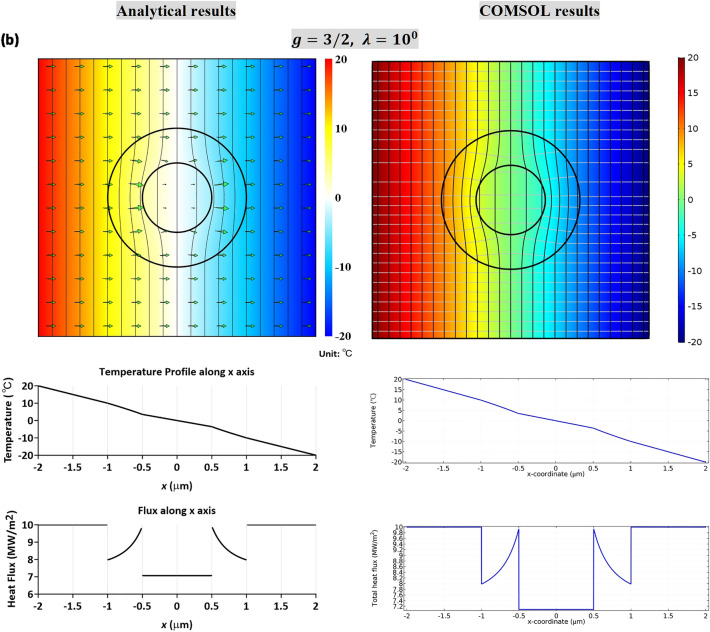
The temperature and heat flux profiles based on the analytical solutions, (6), (7), (9) and (10), and the numerical simulations based on finite element calculations (COMSOL). The left panel is the analytical solutions based on (6) and (7) for LC-type interfaces, and (9) and (10) for HC-type interfaces. The right panel is the numerical simulations based on finite element calculations (COMSOL). In numerical calculations, we consider *c* = 1/4 and *b* = 1 μm. For *g* = 2/3, LC-type interfaces are invoked. Temperature contours for *λ* = 10^0^ is illustrated in (**a**). For *g* = 3/2, HC-type interfaces are necessary, and contour plots for *λ* = 10^0^ are given in (**b**). A temperature or heat flux profile along the *x*-axis is shown below the contour diagram to highlight the jump of relevant quantity across the interface.

In Fig. 5a,b, we plot the temperature and heat flux profiles based on the analytical solutions, (6), (7), (9), (10), and numerical simulations based on finite element calculations (COMSOL). The left panel in Fig. 5 is the analytical solutions based on (6) and (7) for the LC-type interfaces, and (9) and (10)for the HC-type interfaces. The right panel is numerical simulations based on finite element calculations (COMSOL). We mention that the finite element software COMSOL considers that the interfaces are perfect. To simulate the effect of imperfect bonding, we model the imperfect interface as a thin interphase layer of a constant thickness $t$ with isotropic conductivity $k_{C}$, prescribed by either ${{k_{0} t} \mathord{\left/ {\vphantom {{k_{0} t} {a\hat{\beta }_{a} }}} \right. \kern-\nulldelimiterspace} {a\hat{\beta }_{a} }}$(LC-type) or $\hat{\alpha }_{a} ak_{0} /t$ (HC-type). Here we consider a thin thickness *t* = *b*/10^3^ = 10^−3^ μm. In the numerical illustrations, we consider *c* = 1/4, *b* = 1 μm and the value of *λ *= 1. For the LC-type case, we consider *g* = 2/3, while for the HC-type interface we consider *g* = 3/2. To perceive the temperature drop or heat flux drop across the interface, a profile along the *x*-axis is highlighted just below the contour diagram. It is seen that both solutions, analytical solutions and finite-element simulations, agree well. For completeness, additional demonstrations for the temperature contours are presented for *λ* = 10^−1^ and *λ* = 10 in Supplementary Materials, which demonstrate the effect of material anisotropy in Region II. It is seen that the case of *λ* = 10^−1^ will lead to a concentrating effect, while *λ* = 10 will result into a thermal shielding effect. In Supplementary Material Table S1, we also list a comparison of temperature field calculated from finite element numerical simulations, based on COMSOL with three different interphase thickness, *t* = *b*/1200, *t* = *b*/1000 and *t* = *b*/800, and the analytic results for the LC-type interfaces, (6), (7), or for the HC-type interfaces, (9), (10). The percentage error is estimated by (COMSOL prediction − analytic result)/analytic result. We see that the error percentage for *t* = *b*/1200 and *t* = *b*/1000 are nearly the same within 0.3% at most.

## Methods

### Thermal invisibility condition

**Figure 6:**
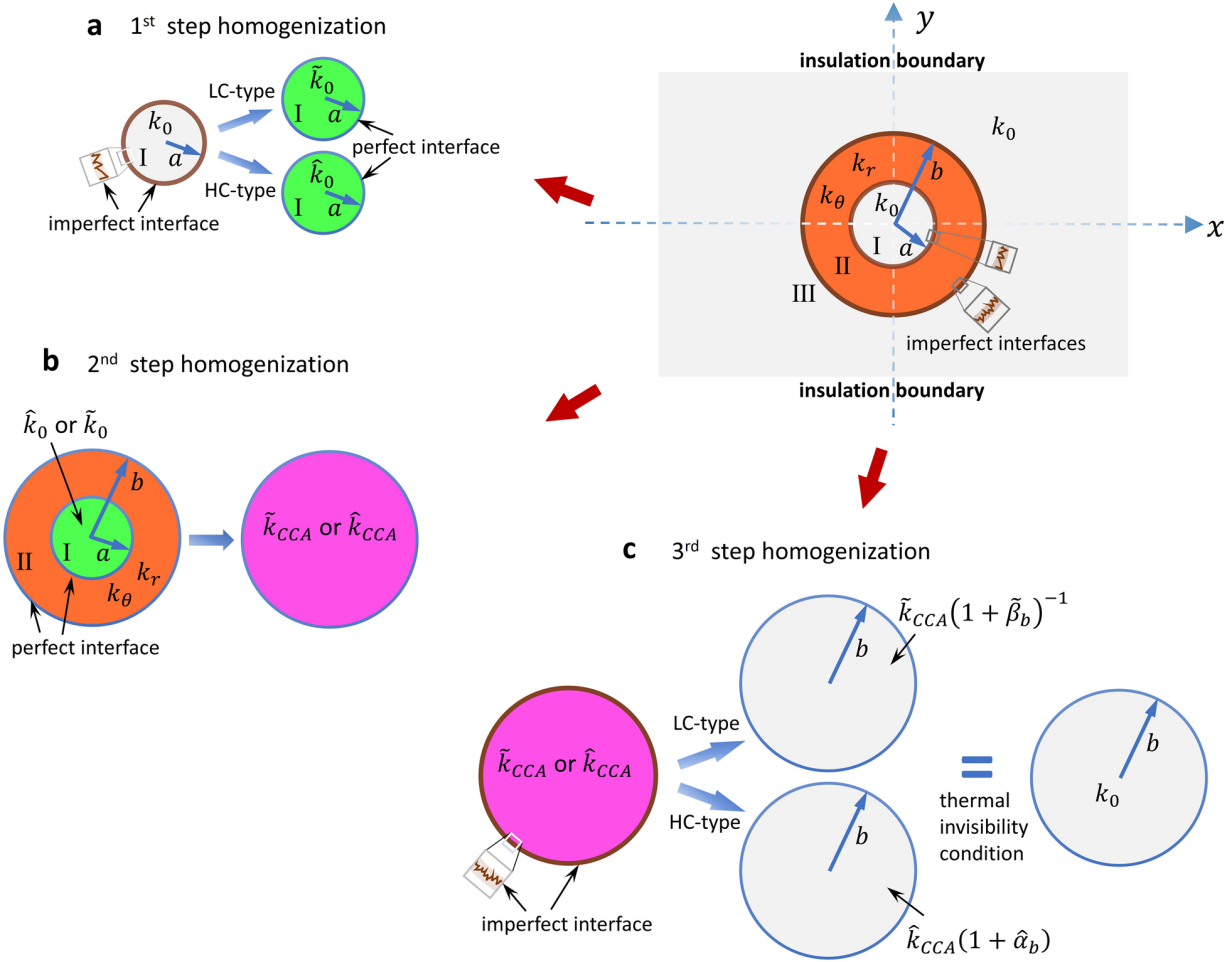
Homogenizations of the coated inclusion with imperfect interfaces. The first step is to consider Region I with the effect of imperfect interface at *r* = *a*. This will give a circular region with an effective isotropic conductivity $\tilde{k}_{0}$ or $\hat{k}_{0}$. Secondly, the composite cylinder model of an isotropic core with conductivity $\tilde{k}_{0}$ or $\hat{k}_{0}$ coated with a cylindrically orthotropic material is determined, expressed as $\tilde{k}_{CCA}$ or $\, \hat{k}_{CCA}$. The final step is to homogenize a uniform conductivity $\tilde{k}_{CCA}$ or $\hat{k}_{CCA}$ with an imperfect effect at *r* = *b*. This will lead to the thermal invisibility condition (3) and (5), respectively.

The thermal invisibility condition (3) for the LC-type interfaces and (5) for the HC-type interfaces can be constructed via the concept of neutral inclusions^32^ through a series of homogenizations via composite cylinder assemblages (CCA)^51^. A schematic illustration is presented in Fig. 6, in which the derivation can be separated into three steps. First, we consider a circular cylinder of radius *a* (Region I) with a LC- or HC-type of boundary surface. The theoretical formulation is to identify an effective conductivity for Region I, without the surface effect, so that the heat flow pattern outside the circular cylinder will be identical for both configurations. The effective conductivity can be exactly derived as ${{\tilde{k}_{0} = \left[ {k_{0}^{ - 1} + \left( {a\beta_{a} } \right)^{ - 1} } \right]^{ - 1} = k_{0} } \mathord{\left/ {\vphantom {{\tilde{k}_{0} = \left[ {k_{0}^{ - 1} + \left( {a\beta_{a} } \right)^{ - 1} } \right]^{ - 1} = k_{0} } {\left( {1 + \tilde{\beta }_{a} } \right)}}} \right. \kern-\nulldelimiterspace} {\left( {1 + \tilde{\beta }_{a} } \right)}}$ for a LC-type interface, and $\hat{k}_{0} = k_{0} + {{\alpha_{a} } \mathord{\left/ {\vphantom {{\alpha_{a} } a}} \right. \kern-\nulldelimiterspace} a} = k_{0} \left( {1 + \hat{\alpha }_{a} } \right)$ for a HC-type interface. Next we consider a circular cylinder with conductivity of either $\tilde{k}_{0}$ or $\hat{k}_{0}$, coated with a circular layer of cylindrically orthotropic material with conductivity tensor (1). This is an exactly solvable composite cylinder assemblage that is amenable to an exact determination for the effective conductivity^51^.

The effective conductivity of composite cylinder assemblages consisting of an isotropic core, with conductivity $\tilde{k}_{0}$ or $\hat{k}_{0} ,$ coated with a cylindrically orthotropic material with conductivity tensor (1) can be expressed as $\tilde{k}_{CCA}$ or $\, \hat{k}_{CCA}$^44^,

11 $$\tilde{k}_{CCA} { = }k_{G} \frac{{\left( {\tilde{g} + 1} \right) + c^{\lambda } \left( {\tilde{g} - 1} \right)}}{{\left( {\tilde{g} + 1} \right) - c^{\lambda } \left( {\tilde{g} - 1} \right)}}{; }\hat{k}_{CCA} { = }k_{G} \frac{{\left( {\hat{g} + 1} \right) + c^{\lambda } \left( {\hat{g} - 1} \right)}}{{\left( {\hat{g} + 1} \right) - c^{\lambda } \left( {\hat{g} - 1} \right)}},$$

where $\tilde{g} = {{\tilde{k}_{0} } \mathord{\left/ {\vphantom {{\tilde{k}_{0} } {k_{G} }}} \right. \kern-\nulldelimiterspace} {k_{G} }}$ and $\hat{g} = {{\hat{k}_{0} } \mathord{\left/ {\vphantom {{\hat{k}_{0} } {k_{G} .}}} \right. \kern-\nulldelimiterspace} {k_{G} .}}$ The last step is to consider a circular cylinder with isotropic conductivity $\tilde{k}_{CCA} {\text{ or }}\hat{k}_{CCA}$ incorporating with the interface effect with surface parameter of $\beta_{b} {\text{ or }}\alpha_{b}$. The analytical result is similar to that in the first step. But now, to be fully compatible to the surrounding Region III, the effective conductivity of the last step is necessarily identical to the surrounding medium, Region III, with conductivity $k_{0} .$ This will lead, respectively, to $\left[ {\tilde{k}_{CCA}^{ - 1} + \left( {b\beta_{b} } \right)^{ - 1} } \right]^{ - 1} = k_{0}$ and $\hat{k}_{CCA} + {{\alpha_{b} } \mathord{\left/ {\vphantom {{\alpha_{b} } b}} \right. \kern-\nulldelimiterspace} b} = k_{0}$, or equivalently,

12 $$\frac{{k_{0} }}{{\tilde{k}_{CCA} }} + \frac{{k_{0} }}{{b\beta_{b} }} = 1,\;\frac{{\hat{k}_{CCA} }}{{k_{0} }} + \frac{{\alpha_{b} }}{{bk_{0} }} = 1.$$

By simple algebra, making use of (11), the thermal invisibility conditions given in (3) and (5) are readily deduced.

### A duality relationship

For a two-dimensional medium, a duality relation is known to exist in the context of conduction phenomena, which links the effective conductivity of a composite to that of a composite with the same phase geometry but replacing its phase moduli with the inverse conductivities^32,52–54^. The existence of the connection between the dual systems relies on the fact that a two-dimensional divergence free field, when rotated pointwise by a 90 degree, produces a curl free field, and vice versa. Lipton^45^ proved that the duality relation is also applicable to a composite system with imperfect interfaces. Here we demonstrate that our invisibility conditions (3) and (5) can indeed be linked with each other via the duality relation. For simplicity of expositions, we will make use of Proposition 2 given in the work^55^, recorded as $k^{*\beta } \left( {k_{1} ,k_{2} , \ldots ,k_{n} ,\beta } \right) = {1 \mathord{\left/ {\vphantom {1 {k^{*\alpha } \left( {k_{1}^{ - 1} ,k_{2}^{ - 1} , \ldots ,k_{n}^{ - 1} ,\alpha^{ - 1} } \right)}}} \right. \kern-\nulldelimiterspace} {k^{*\alpha } \left( {k_{1}^{ - 1} ,k_{2}^{ - 1} , \ldots ,k_{n}^{ - 1} ,\alpha^{ - 1} } \right)}}.$ Here the superscript $k^{*\beta }$ represents the effective conductivity of the composite system with LC-type interfaces, while $k^{*\alpha }$ is the effective conductivity of the dual system with HC-type interfaces. The arguments inside the parenthesis of $k^{*\alpha }$ or $k^{*\beta }$ are simply the phase conductivities and the interface parameters of the composite system. Now letting $k_{0} \to k_{0}^{ - 1}$, $\tilde{k}_{CCA}^{ - 1} \to \hat{k}_{CCA}$ and $\beta^{ - 1} \to \alpha$ in (12)_1_, we can see that (12)_2_ is readily deduced. Likewise, one can deduce (12)_1_ from (12)_2_. We thus conclude that the system with LC- and HC-type interfaces are complementary with each other, that provides a link to achieve thermal invisibility for the case of *k*_*G*_ > *k*_0_ or *k*_*G*_ < *k*_0_.

## Conclusions

Utilizing the theory of neutral inclusions in mechanics of composites, we provide a thorough analysis on the effect of imperfect interfaces in functional thermal metamaterials, made of homogeneous anisotropic conductivity. When the interfaces are perfectly bonded, the condition of $k_{0}^{2} = k_{r} k_{\theta }$ will ensure that the temperature field in Region III will be the same as that of a homogeneous background material. While for interfaces, the neutrality conditions can be constructed in the well-structured forms (3) and (5). Specifically, they depend on the absolute size, the material anisotropy $\lambda$ and the area fraction $c.$ This finding now works at new levels of applicability, irrespective of the bonding perfectness $g$. In general, the imperfect interface will broadly affect the heat transfer across interface and will exhibit size effect. We demonstrate quantitatively the interplay between the material and geometric parameters of the configuration, and also explore the extent to which a thermal confinement or thermal shielding can be actually achieved within a targeted region. Numerical simulations based on finite element calculations are also carried out to validate our analytical results. We prove that the thermal invisibility conditions with the LC- and HC-type interfaces are reciprocal to each other which, all together, will span a full spectrum for the feasibility of thermal invisibility. We believe that the study provides further insights to the understanding of heat transfer mechanisms across interfaces in the performances of thermal metamaterials, and will motivate future experiments for practical realization. Since the interface in general may not be always perfect, this finding can serve as a general guideline in the design of thermal metamaterials that facilitates broad applications.

## Supplementary Information

Supplementary Information.
