# Supplementary material for: Novel connections and physical implications of thermal metamaterials with imperfect interfaces
Source: Sci Rep. 2022 Feb 17;12:2734. doi: 10.1038/s41598-022-06719-1 (PMC8854668; doi:10.1038/s41598-022-06719-1)
Supplement: Supplementary file 1 — Supplementary Information. [file 41598_2022_6719_MOESM1_ESM.docx]

Supplementary Materials

Novel Connections and Physical Implications of

Thermal Metamaterials with Imperfect Interfaces

**Tungyang Chen*, and Jun-Hong Lin**

Department of Civil Engineering, National Cheng Kung University

Tainan, 70101, Taiwan, *^*^*Corresponding author: [tchen@mail.ncku.edu.tw](mailto:tchen@mail.ncku.edu.tw)

In this Supplementary Information, we provide details for the derivation of temperature field for the configuration (Figure 1) under the thermal invisibility conditions in (3) or (5). The governing equation for the temperature field in Regions I and III is simply the Laplace equation, while in Region II the temperature field equation can be written as

$\frac{\partial^{2}T}{\partial r^{2}}+\frac{1}{r}\frac{\partial T}{\partial r}+\frac{\lambda^{2}}{r^{2}}\frac{\partial^{2}T}{\partial\theta^{2}}=0,$ (S1)

with $\lambda$ being defined in the main text. Suppose that temperature boundary conditions $T\left(x=r\cos\theta=\pm x_{0}\right)=\mp T_{0}$ are prescribed on the left and right surfaces of the configuration of Figure 1, where $T_{0}=-Ex_{0}$ and *E* is the prescribed constant thermal intensity. Taking into account the boundary condition and the symmetry condition of *T*, the temperature field in the three regions can be expressed as

$$T_{1}\left(r,\theta\right)=A_{1}r\cos\theta,$$

$$r\leq a$$

$T_{2}\left(r,\theta\right)=\left(A_{2}r^{\lambda}+B_{2}r^{-\lambda}\right)\cos\theta,$ $a\leq r\leq b$ (S2)

$$T_{3}\left(r,\theta\right)=\left(A_{3}r+B_{3}r^{-1}\right)\cos\theta,$$

$$r\geq b.$$

The boundary condition at $x=\pm x_{0}$ will give readily$A_{3}=-E$ and the coefficients *A*_1_, *A*_2_, *B*_2_, and *B*_3_ in (S2) will be determined from by the LC-type interface conditions,

$k_{r}\frac{\partial T_{2}}{\partial n}=k_{0}\frac{\partial T_{1}}{\partial n}\left.=\beta_{a}\left(T_{2}-T_{1}\right)\right|_{r=a},$ $k_{0}\frac{\partial T_{3}}{\partial n}=k_{r}\frac{\partial T_{2}}{\partial n}=\left.\beta_{b}\left(T_{3}-T_{2}\right)\right|_{r=b}.$ (S3)

or from the HC-type interface conditions,

| $\left.T_{1}=T_{2}\right\vert_{r=a},$ | $\left.\left(k_{0}\frac{\partial T_{1}}{\partial r}-k_{r}\frac{\partial T_{2}}{\partial r}\right)\right\vert_{r=a}=\left.\left(\alpha_{a}\Delta_{s}T\right)\right\vert_{r=a},$ | (S4) |
| --- | --- | --- |
| $\left.T_{3}=T_{2}\right\vert_{r=b},$ | $\left.\left(k_{r}\frac{\partial T_{2}}{\partial r}-k_{0}\frac{\partial T_{3}}{\partial r}\right)\right\vert_{r=b}=\left.\left(\alpha_{b}\Delta_{s}T\right)\right\vert_{r=b}$. |

Here $\Delta_{s}$is the surface Laplacian operator. Note that we are aiming to devise a metamaterial layer with constant anisotropic conductivity tensor so that the outer field in Region III will remain to have a constant temperature intensity *E*, as if the whole medium is spatially uniform. In addition, the thermal intensity in Region I can be tuned to be greatly enhanced or shielded. As the outer region will have a uniform temperature gradient *E*, this implies that the constant $B_{3}$ must be zero. As a result, we have four interface conditions to be fulfilled from three unknown coefficients, *A*_1_, *A*_2_, and *B*_2_. This will lead to an overdetermined system of algebraic equations. The condition for the existence of the solutions, *A*_1_, *A*_2_, and *B*_2_ will lead to the thermal invisibility conditions. The derivation will involve three interface conditions to solve for the three unknown coefficients and utilize the solutions for the coefficients into the fourth condition (the remaining interface condition). This will provide the thermal invisibility conditions given in (3) and (5). A direct algebra will be complicated and somewhat awkward. In Section 4 in the main text, we have presented a systematic procedure through a series of composite cylinder assemblages in a simple manner. We have analytically verified that both approaches are analytically equivalent.

Now let us define$\Lambda=\left(1+\hat{\beta}_{a}\right)/g$, the thermal invisibility condition (3) can be recast as

$\frac{\Lambda-\tanh\left(\frac{\lambda}{2}\ln c\right)}{1-\Lambda\tanh\left(\frac{\lambda}{2}\ln c\right)}=\frac{1-\hat{\beta}_{b}}{g}.$ (S5)

For a fixed value of *c*, we plot in **Figure 4** the value of *T*_1_*/T*_3_ versus *λ* for different values of Λ. Likewise, one can define $\Gamma=g\left(1+\hat{\alpha}_{a}\right)$ and rewrite (5) as

$\frac{\Gamma-\tanh\left(\frac{\lambda}{2}\ln c\right)}{1-\Gamma\tanh\left(\frac{\lambda}{2}\ln c\right)}=\left(1-\hat{\alpha}_{b}\right)g.$ (S6)

The thermal invisibility conditions are listed in Eq. (3) in the main text for the LC-type and in Eq. (5) for the HC type interfaces. Herr we provide analytical discussions on the effects of extreme values of $\lambda$, the effect of different values of *g = k*_0_ */ k_G_* and the effect of different values of *c* = (*a / b*)^2^.

**The effect of extreme values of** $\lambda=\sqrt{k_{\theta}/k_{r}}$

We consider the extreme situations of *λ *→ ∞ and *λ *→ 0, which will correspond respectively to $k_{\theta}\gg k_{r}$ and $k_{r}\gg k_{\theta}.$ First, for *λ *→ ∞**,** from (3) and (5), simple algebra will show that$g+\tilde{\beta}_{b}=1$for$g<1$, and$\text{ }g^{-1}+\hat{\alpha}_{b}=1$ for *g* > 1. It can be seen that both conditions are independent of the area fraction *c*, and that non-dimensional parameters $\tilde{\beta}_{b}$ and $\hat{\alpha}_{b}$ are non-negative, as they should be. Physically, for *λ *→ ∞ the heat flow will be guided around the Region II circumferentially and thus the thermal invisibility condition will be independent of the area fraction *c*. Next, for *λ *→ 0, we have$c^{\lambda}\to1$. It can be seen that in this case, to fulfill the invisibility requirement in (3) and (5), we must have perfect bonding interfaces, namely $\hat{\alpha}_{a}=\hat{\alpha}_{b}=0$ and $\tilde{\beta}_{a}=\tilde{\beta}_{b}=0$.

The effect of different values of *g = k*_0_ */ k_G_*

The parameter of *g* highlights the relative magnitude of *k_G_* and *k*_0_. When *g* → 10^0^ =1, by (3) and (5), we know that *k_G_* = *k*_0_ is the invisibility condition for perfect bonding interfaces, that is $\hat{\alpha}_{a}=\hat{\alpha}_{a}=0$ and $\hat{\beta}_{a}=\hat{\beta}_{b}=0$. When the value of *g* starts to deviate from the value of unity, the values of the interface parameters $\hat{\alpha}$ or $\hat{\beta}$ will be increasing from zero to compensate the discrepancy between *k_G_* and *k*_0_. When *g* → 0, that is $k_{G}\gg k_{0}$**,** one can see from (3) that $\hat{\beta}_{b}\to1.$ Physically, this represents an “adiabatic” boundary condition^34^. Also, when *g* → ∞, that is $k_{G}\ll k_{0}$**,** from (5) we are led to $\hat{\alpha}_{b}\to1.$ The limiting case of $\hat{\alpha}_{b}\to1$ represents the situation that the interface is infinitely superconducting^47^.

The effect of different values of *c* = (*a / b*)^2^

The value of *c* represents the area fraction of Region I within the circular region$r\leq b$. When *c*→ 0, Region I will shrink to a point and Region II becomes a circle with radius *b*, composed of cylindrically orthotropic material. From (3) and (5), we have $g+\tilde{\beta}_{b}=1$ for *g* < 1, and $\text{ }g^{-1}+\hat{\alpha}_{b}=1$ for *g* > 1. By direct expansion, we find that there exists a critical radius of *b* such that the inclusion can become thermally invisible,$b_{cr}=\beta_{b}^{-1}/\left(k_{0}^{-1}-k_{G}^{-1}\right)$ for *g* < 1 and $b_{cr}=\alpha_{b}/\left(k_{0}-k_{G}\right)$ for *g* > 1. When Region II is isotropic, these results recover the previously known results that an isotropic inclusion together with imperfect interface can be made neutral ^51^. We note also that, under the condition of *c *→ 0, the result is identical to the case that we have derived earlier for *λ *→ ∞. When *c *→ 1, from (3) and (5), by taking a limiting process, it can be verified that to fulfill the invisibility condition we must have perfect bonding interfaces, that is $\hat{\alpha}_{a}=\hat{\alpha}_{a}=0$ or $\tilde{\beta}_{a}=\tilde{\beta}_{b}=0$, where the whole medium becomes homogenous throughout with an isotropic conductivity *k*_0_.

In Figure (S1), we present the temperature and heat flux profiles for *λ *= 10^−1^ and *λ *= 10 based on the analytical solutions, (6), (7), (9), and (10), and the numerical simulations based on finite element calculations (COMSOL). The left panel in Figure (S1) is the analytical solutions based on (6) and (7) for the LC-type interfaces, and (9) and (10) for the HC-type interfaces. For *g* = 2/3, temperature contours for two different values of *λ *= 10^−1^ and *λ *= 10 are illustrated in (a), and (b), respectively. For *g* = 3/2, a HC-type interface is invoked, and contours plots for *λ *= 10^−1^ and *λ *= 10 are given in (c) and (d) respectively. The right panel is the numerical simulations based on finite element calculations (COMSOL). As in Figure 5, we consider *c* = 1/4, *b =* 1 *μm*. For the LC-type interface, we consider *g* = 2/3, while for the HC-type interface we consider *g* = 3/2. It is seen that the case of *λ *= 10^−1^ will lead to a concentrating effect, while *λ *= 10 will result into a thermal shielding.

In the finite element simulations based on COMSOL in Figure 5 and **Figure S1**, we have assume a thin interphase of constant thickness *t* = *b*/10^3^ = 10^-3^ *μm*. See a schematic illustration in **Figure S2** for the thickness *t* of the interphase layer. In **Table S1**, we list a comparison of temperature fields based on numerical simulations, with three different interphase thickness, *t* = *b* / 1200, *t* = *b* / 1000 and *t* = *b* / 800. The analytic results are based on Eqs. (6) and (7) for the LC-type interfaces, and Eqs. (9) and (10) for the HC-type interfaces. The percentage error is evaluated by (COMSOL prediction – analytic result)/analytic result. We see that the error percentage for *t* = *b* / 1200 and *t* = *b* / 1000 are nearly the same within $0.3\%$ at most. Therefore, for the finite element calculations, we select *t* = *b* / 1000 in our calculations.

| **Analytical results** | **COMSOL results** |
| --- | --- |
| 1. **,** | |
|  | 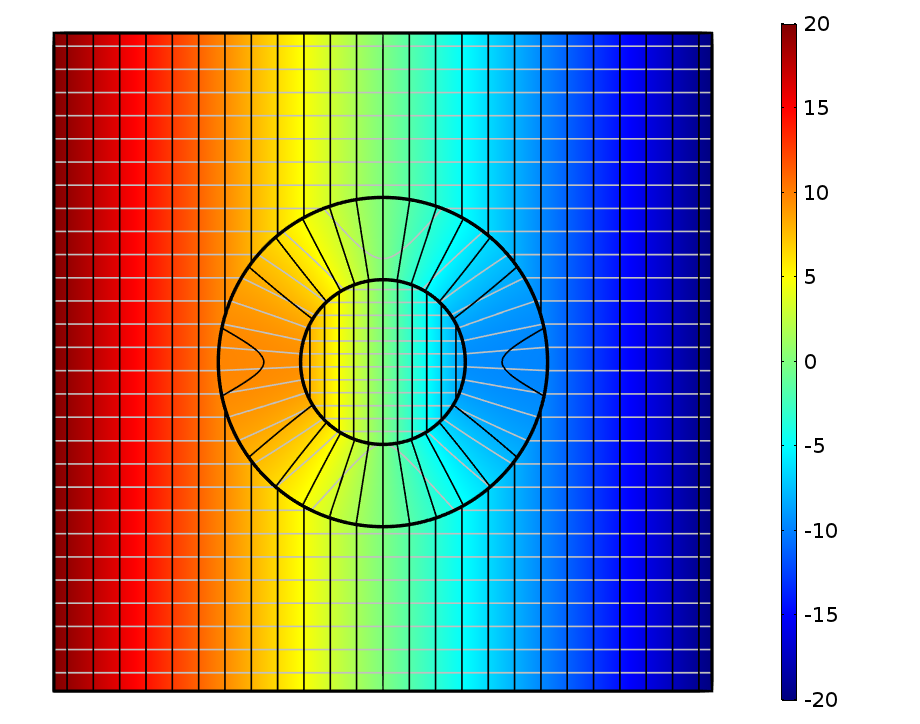  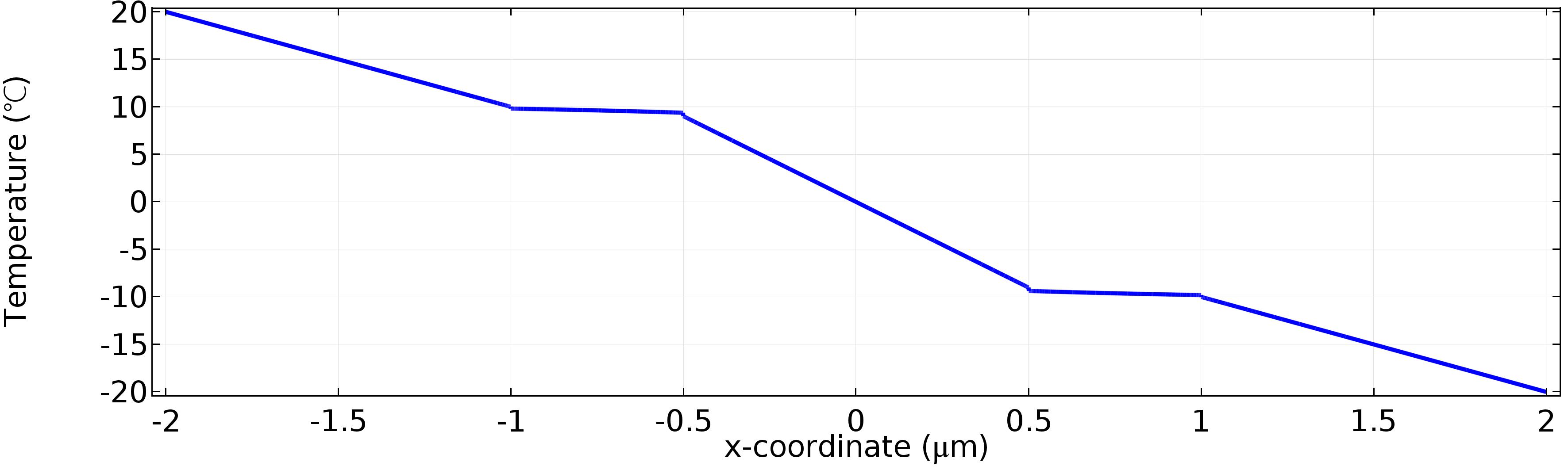  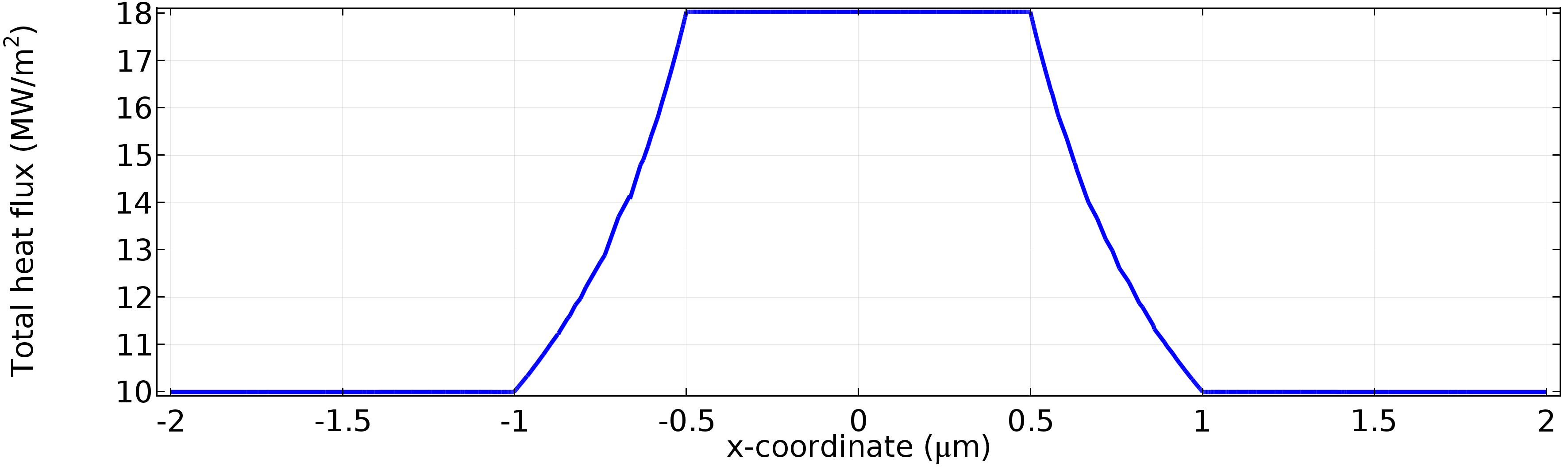 |

| **Analytical results** | **COMSOL results** |
| --- | --- |
| 1. **,** | |
|  | 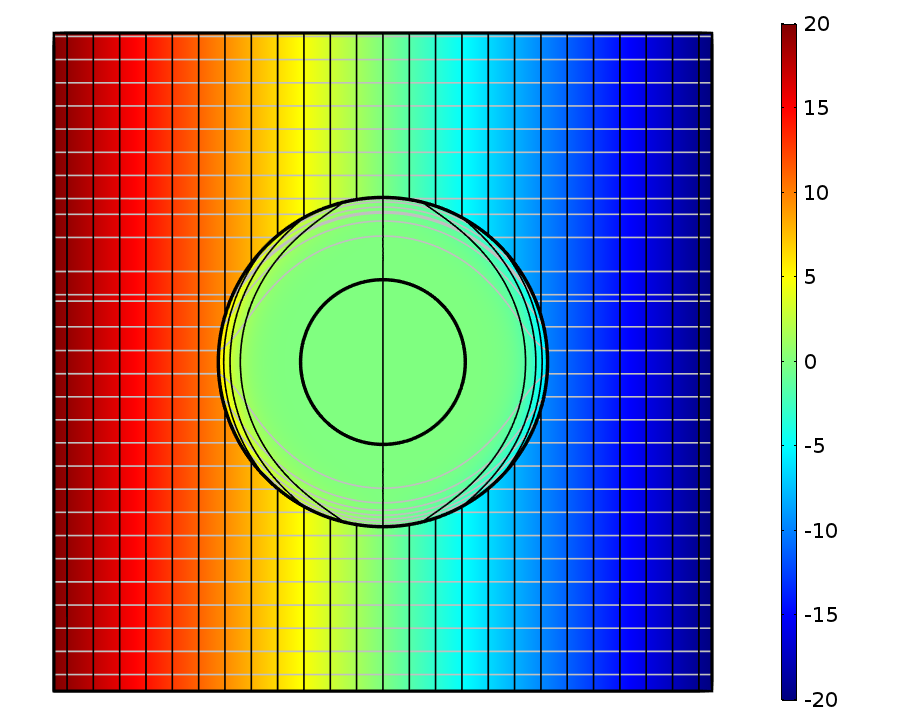  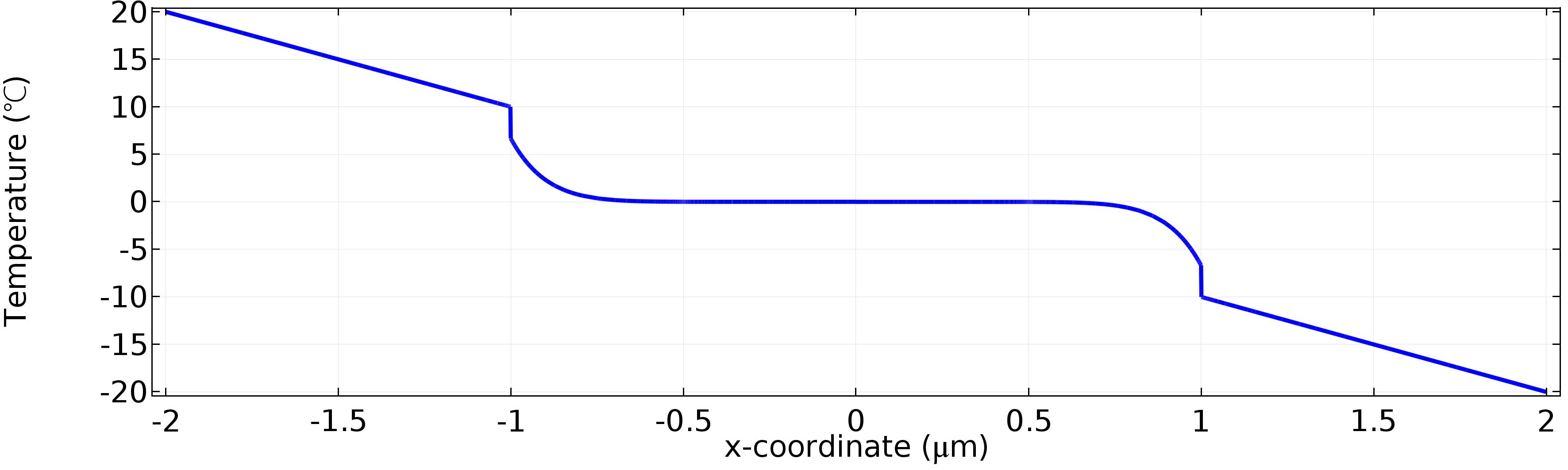  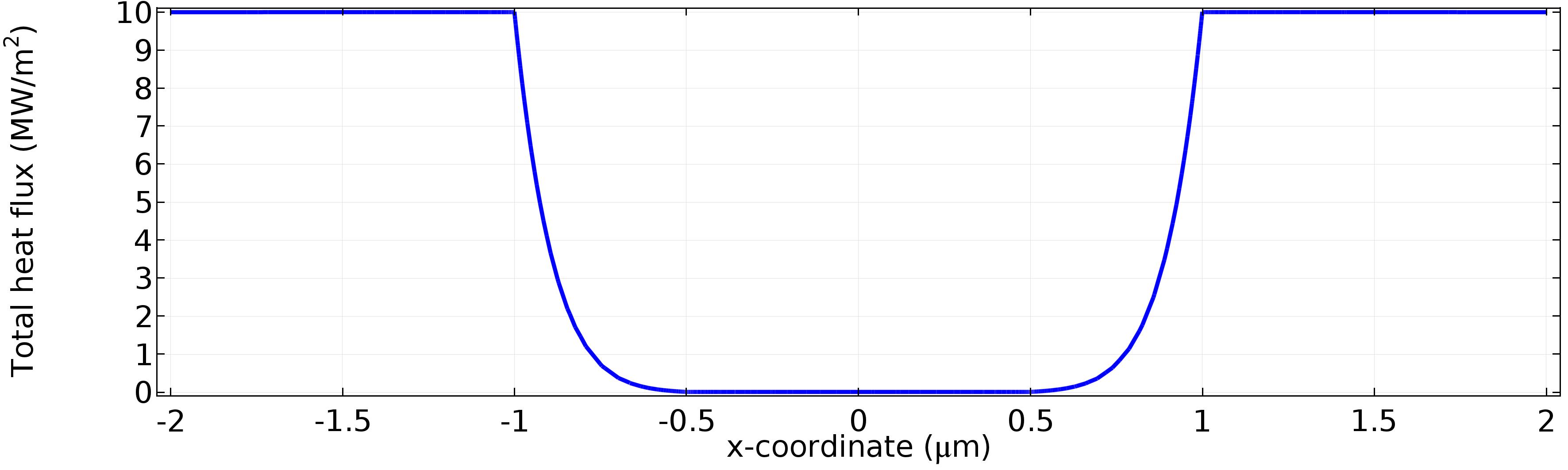 |

| **Analytical results** | **COMSOL results** |
| --- | --- |
| 1. **,** | |
|  | 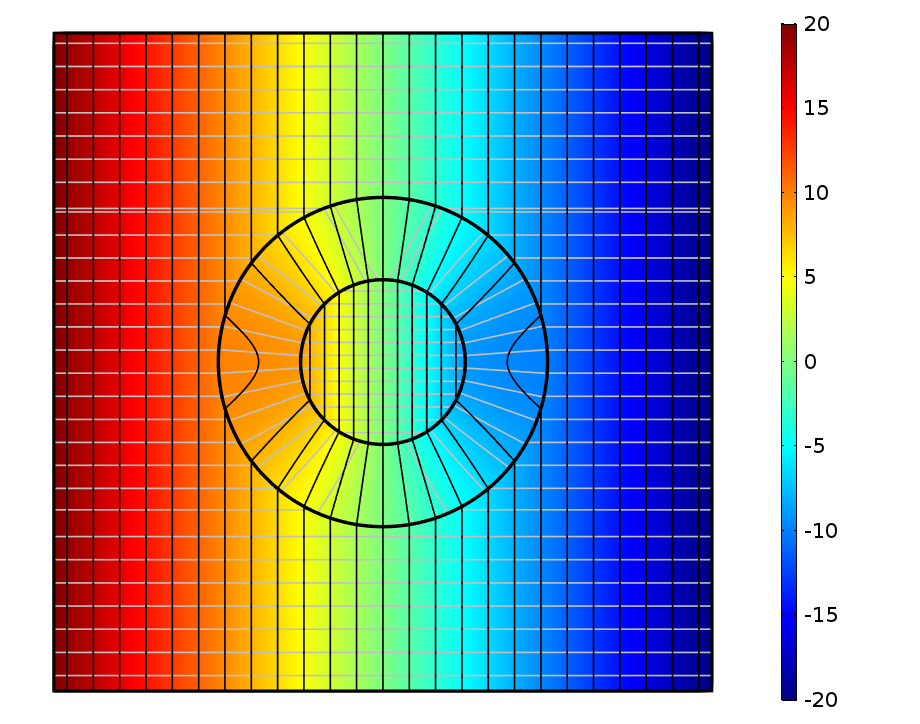  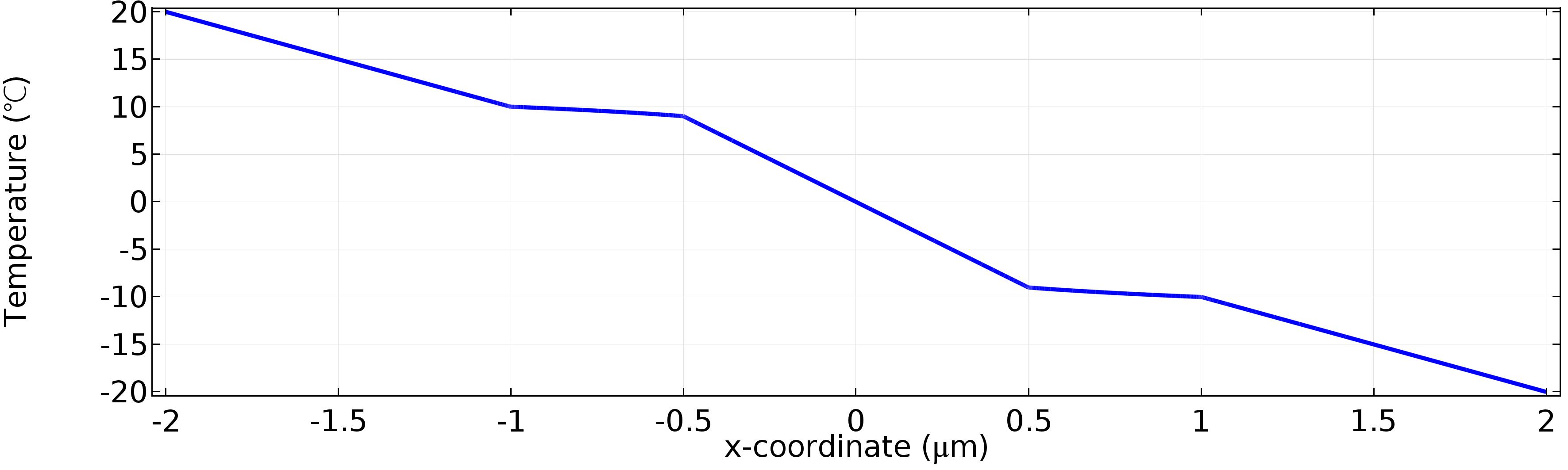  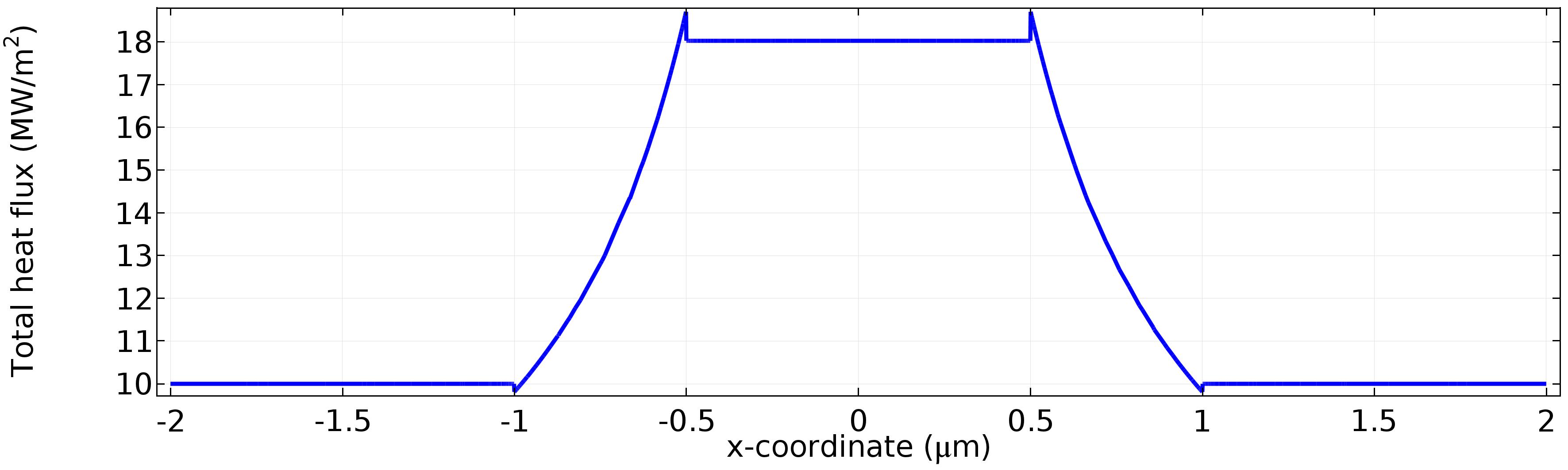 |

| **Analytical results** | **COMSOL results** |
| --- | --- |
| 1. **,** | |
|  | 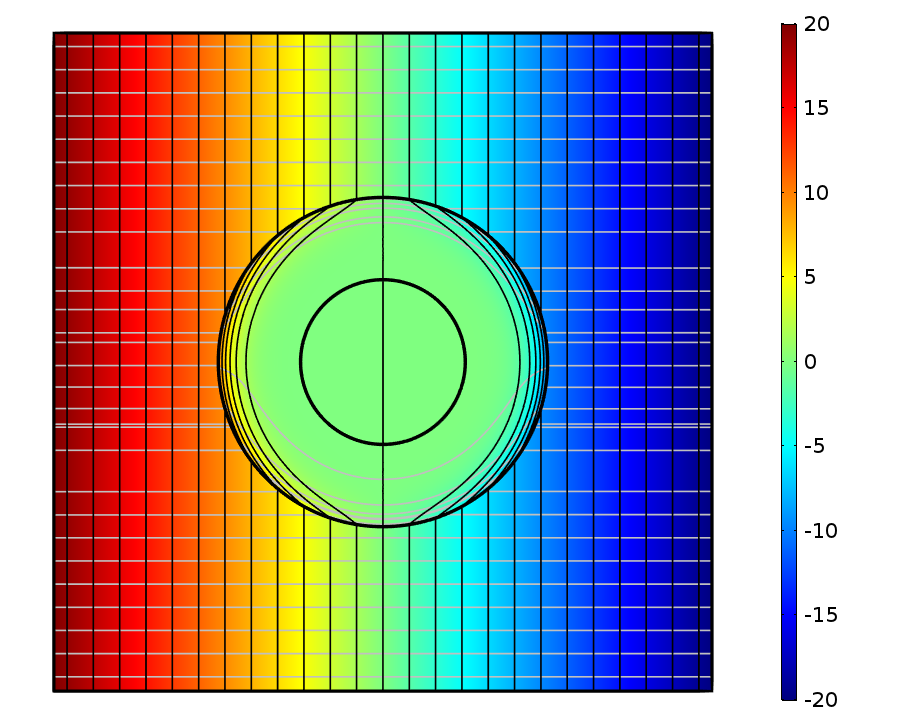  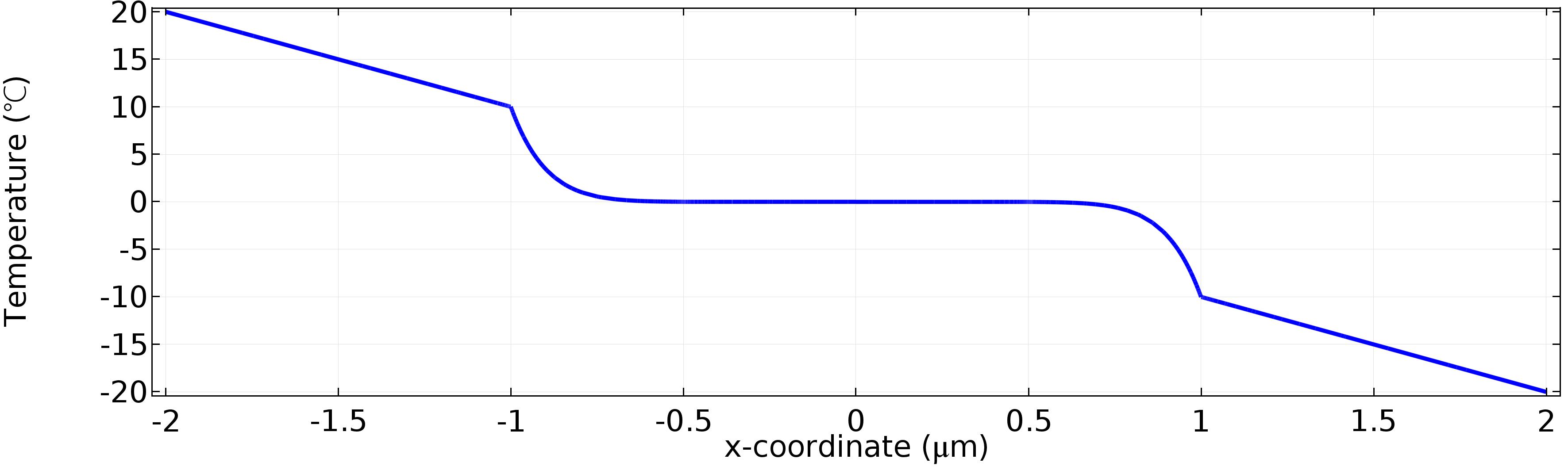  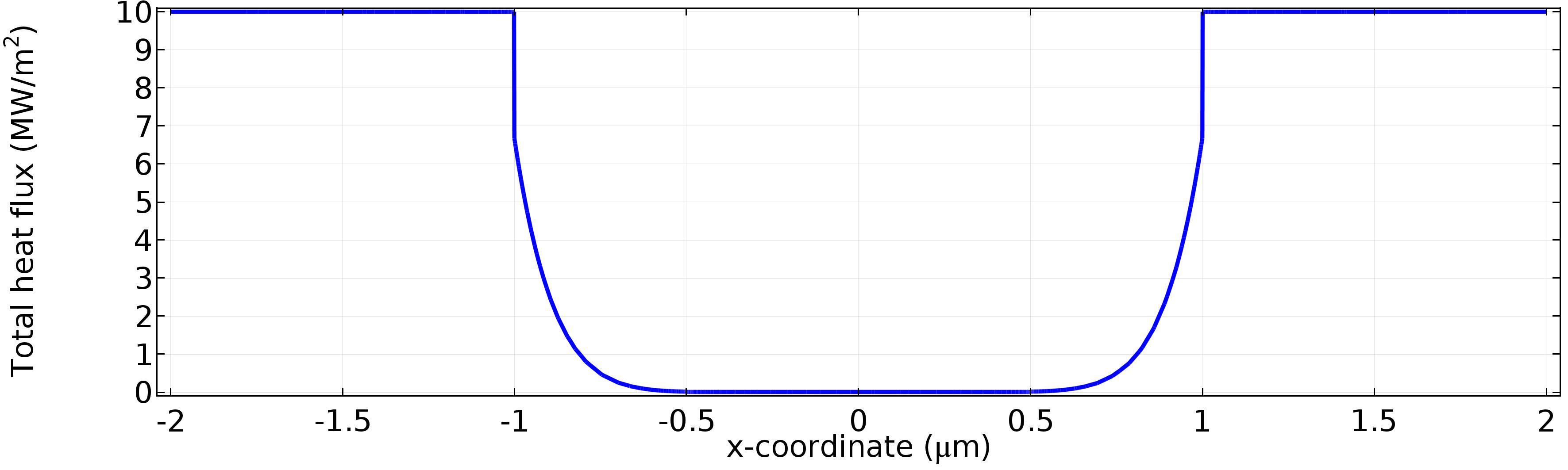 |

**Figure S1**. The temperature and heat flux profiles based on the analytical solutions, (6) and (9), and the numerical simulations based on finite element calculations (COMSOL). The left panel is the analytical solutions based on (6) and (7) for LC-type interfaces, and (9) and (10) for HC-type interfaces. The right panel is numerical simulations based on finite element calculations (COMSOL). In numerical calculations, we consider *c* = 1/4 and *b =* 1 *μm*. For *g* = 2/3, LC-type of interfaces are invoked. Temperature contours for two different values of *λ *= 10^−1^ and *λ *= 10 are illustrated in (a), and (b) respectively. For *g* = 3/2, HC-type interfaces are necessary, and contours plots for *λ *= 10^−1^ and *λ *= 10 are given in (c) and (d), respectively. A temperature or heat flux profile along the *x*-axis is shown below the contour diagram to highlight the discontinuity of relevant quantity across the interface.


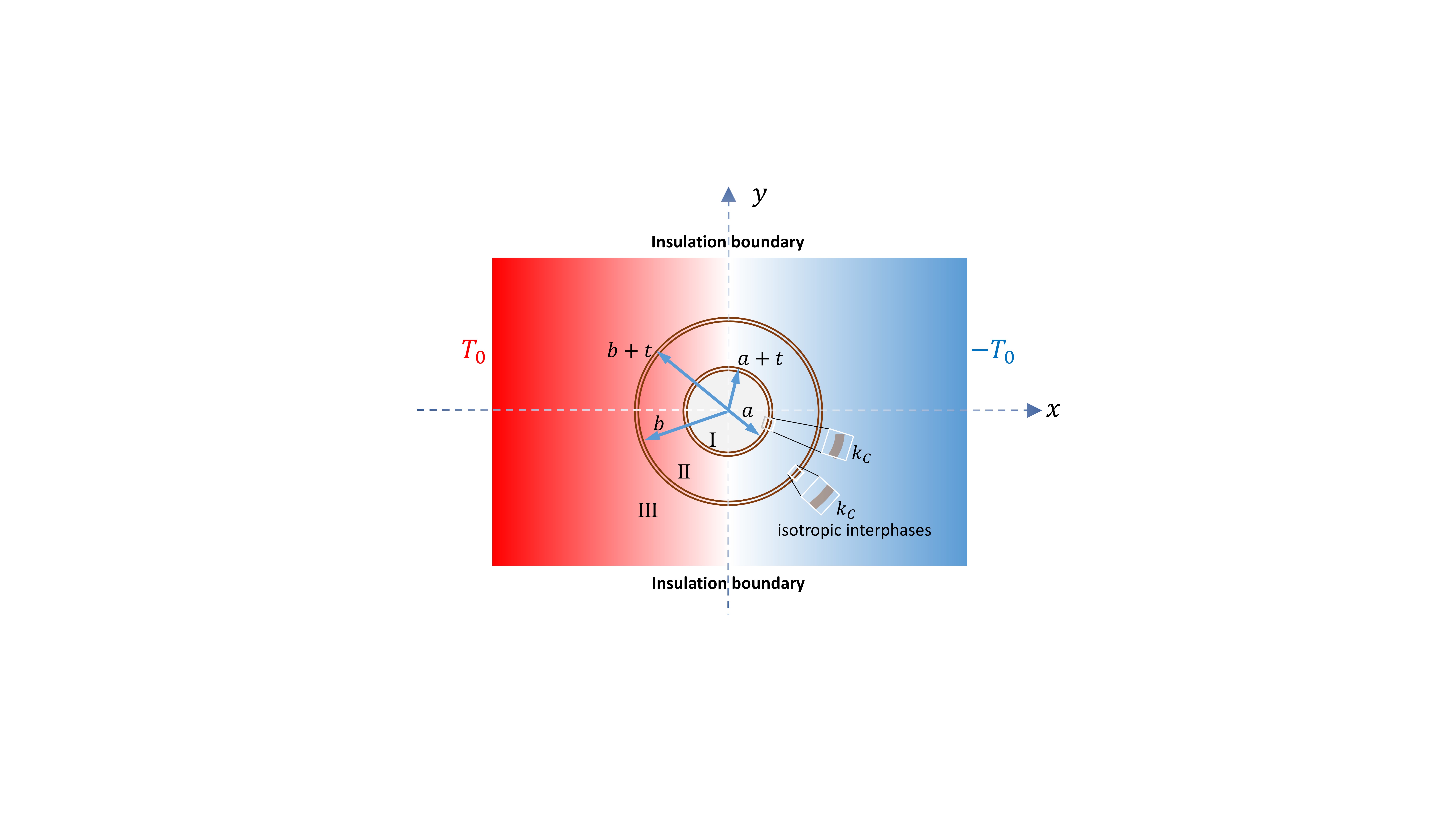


**Figure S2.** A schematic illustration of the thermal device established in COMSOL. The imperfect interfaces at *r = a* and *r = b* are both simulated by ultrathin layers with thickness $t$ and isotropic conductivity *k_c_*. Theoretically the thickness *t* approaches to zero in comparison with the geometry size. These two coating layers ranges respectively from *a* to *a+t* and from *b* to *b+t*. The conductivity *k_c_* is determined by the invisibility condition. Here we consider *a =* 0.5 *μm* and *b =* 1 *μm* in COMSOL simulations.

**Table S1**. Comparison of the numerical results based on COMSOL simulations

and the analytical solution

1. $\boldsymbol{g}\mathbf{=2/3}$**, $\boldsymbol{\lambda}\mathbf{=}\mathbf{10}^{\mathbf{0}}$ (LC-type)**

| analytic | coordinate | $x=-b^{-}$ | $x=-b^{+}$ | $x=-a^{-}$ | $x=-a^{+}$ | $x=a^{-}$ | $x=a^{+}$ | $x=b^{-}$ | $x=b^{+}$ |
| --- | --- | --- | --- | --- | --- | --- | --- | --- | --- |
|  | T (°C) | 10 | 7.9717 | 4.9646 | 3.531 | -3.5319 | -4.9646 | -7.9717 | -10 |
| COMSOL | coordinate | $x=-b-t$ | $x=-b$ | $x=-a-t$ | $x=-a$ | $x=a$ | $x=a+t$ | $x=b$ | $x=b+t$ |
| *t*=  *b*/1200 | T (°C) | 10.01 | 7.981 | 4.9771 | 3.5416 | -3.5416 | -4.9771 | -7.981 | -10.01 |
|  | error (%) | 0.100% | 0.117% | 0.252% | 0.300% | 0.275% | 0.252% | 0.117% | 0.100% |
| *t*=  *b*/1000 | T (°C) | 10.012 | 7.9828 | 4.9795 | 3.5435 | -3.5435 | -4.9795 | -7.9828 | -10.012 |
|  | error (%) | 0.120% | 0.139% | 0.300% | 0.354% | 0.328% | 0.300% | 0.139% | 0.120% |
| *t*=*b*/800 | T (°C) | 9.609 | 7.9856 | 4.6962 | 3.5464 | -3.5464 | -4.6962 | -7.9856 | -9.609 |
|  | error (%) | -3.910% | 0.174% | -5.406% | 0.436% | 0.411% | -5.406% | 0.174% | -3.910% |

T: temperature in °C

1. $\boldsymbol{g}\mathbf{=3/2}$**, $\boldsymbol{\lambda}\mathbf{=}\mathbf{10}^{\mathbf{0}}$ (HC-type)**

| analytic | coordinate | $x=-b^{-}$ | $x=-b^{+}$ | $x=-a^{-}$ | $x=-a^{+}$ | $x=a^{-}$ | $x=a^{+}$ | $x=b^{-}$ | $x=b^{+}$ |
| --- | --- | --- | --- | --- | --- | --- | --- | --- | --- |
|  | heat flux | 10 | 7.97168 | 9.92921 | 7.06371 | 7.06371 | 9.92921 | 7.97168 | 10 |
| COMSOL | coordinate | $x=-b-t$ | $x=-b$ | $x=-a-t$ | $x=-a$ | $x=a$ | $x=a+t$ | $x=b$ | $x=b+t$ |
| *t*=  *b*/1200 | heat flux | 10.002 | 7.9816 | 9.9382 | 7.0836 | 7.0848 | 9.9382 | 7.9807 | 10.002 |
|  | error (%) | 0.020% | 0.124% | 0.091% | 0.282% | 0.299% | 0.091% | 0.113% | 0.020% |
| *t*=  *b*/1000 | heat flux | 10.003 | 7.9835 | 9.94 | 7.0876 | 7.089 | 9.94 | 7.9826 | 10.003 |
|  | error (%) | 0.030% | 0.148% | 0.109% | 0.338% | 0.358% | 0.109% | 0.137% | 0.030% |
| *t*=*b*/800 | heat flux | 9.6003 | 7.9865 | 9.3741 | 7.0935 | 7.0855 | 9.3741 | 7.9836 | 9.6003 |
|  | error (%) | -3.997% | 0.186% | -5.591% | 0.422% | 0.308% | -5.591% | 0.150% | -3.997% |

heat flux: MW⋅m^-2^

The symbol *t* denotes the thickness of the interphase, and error (%), which is given by (COMSOL-analytic)/analytic, indicates the percent error between the COMSOL calculation and the analytical solution.
